# Therapeutic Applications of Human and Bovine Colostrum in the Treatment of Gastrointestinal Diseases and Distinctive Cancer Types: The Current Evidence

**DOI:** 10.3389/fphar.2020.01100

**Published:** 2020-09-11

**Authors:** Siddhi Bagwe-Parab, Pratik Yadav, Ginpreet Kaur, Hardeep Singh Tuli, Harpal Singh Buttar

**Affiliations:** ^1^ Shobhaben Pratapbhai Patel School of Pharmacy and Technology Management, Shri Vile Parle Kelavani Mandals Narsee Monjee Institute of Management Studies, Mumbai, India; ^2^ Department of Biotechnology, Maharishi Markandeshwar (Deemed to be University), Mullana, Ambala, India; ^3^ Department of Pathology and Laboratory Medicine, Faculty of Medicine, University of Ottawa, Ottawa, ON, Canada

**Keywords:** inflammatory bowel disease (IBD), anticancer therapy, antimicrobial activity, cervical intraepithelial neoplasia, proline-rich polypeptide, immunoglobulins, conjugated linoleic acid

## Abstract

The incidence of gastrointestinal disorders (GID) and cancers is escalating all over the world. Limited consumption of colostrum by newborns not only weakens the immune system but also predisposes infants to microbial infections. Colostrum is nature’s perfect food, sometimes referred to as the ‘elixir of life’. Breast-fed infants have a lower incidence of GI tract infections than infants fed formula or cow’s milk. As per WHO statistics, cancer is the most prevalent disease globally and causes 9.6 million deaths worldwide. The current strategies for treating cancer include chemotherapy, radiation, and surgery. However, chemotherapy and radiation exposure are usually associated with serious long-term side effects and deterioration in the quality of life (QOL) of patients. Furthermore, the hospitalization and medication costs for treating cancers are exorbitant and impose high economic burden on healthcare systems. People are desperately looking for cost-effective and affordable alternative therapies for treating GID and cancers. Therefore, there is an urgent need for clinically evaluating the anticancer compounds isolated from plants and animals. Such therapies would not only be economical and have fewer side effects, but also help to improve the QOL of cancer patients. Recently, bovine colostrum (BC) has caught the attention of many investigators to explore its anticancer potential in humans. BC impregnated dressings are highly effective in treating chronic wounds and diabetic foot ulcer. BC is rich in lactoferrin, a glycoprotein with strong antioxidant, anti-inflammatory, anti-cancer, and anti-microbial properties. Intravaginal application of BC tablets is effective in causing the regression of low-grade cervical intraepithelial neoplasia. The underlying mechanisms of BC at cellular, genetic, and molecular levels remain to be ascertained. Oral BC supplement is well-tolerated, but some people may experience problems such as flatulence and nausea. Well-designed, randomized, placebo-controlled, clinical trials are needed to access the therapeutic potential, long-term safety, and optimal doses of BC products. This review is aimed to highlight the anticancer potential of BC and its components, and the therapeutic applications of BC supplements in treating gastrointestinal diseases in children and adults. We also discuss the health promotion benefits and therapeutic potential of BC nutraceuticals in reducing the incidence of non-communicable diseases.

## Introduction

### Role of Colostrum Against Gastrointestinal Disorders (GIDs)

Necrotizing enterocolitis and neonatal sepsis are the major gastrointestinal ailments in premature babies, newborns, and toddlers, especially those whose mothers are unable to provide colostrum. Breast-fed infants have a lower incidence of GI tract infections than infants fed with formula or cow’s milk. GIDs can lead to stunted physical growth and neurodevelopment, retarded immune function, malabsorption of nutrients, and susceptibility to other diseases like allergies and asthma at an early age. In newborns, colostrum acts as a broad-spectrum antibacterial agent that protects against gut infections as well as contributes to physical growth, immune function, and development of the GI tract. In adults, colostrum promotes healing of the GI tract and protects against gut pathogens (bacteria, viruses, fungi, yeast, mold, etc.), and leaky gut syndrome. Mother’s colostrum or first milk is significantly richer in biologically active peptides, anti-oxidants, anti-inflammation agents, and growth promoting factors that differ substantially from later milk (Bagwe et al., 2015; Buttar et al., 2017). According to Gensollen et al. (2016), some GIDs are caused by the compromised immune system in neonates. The intake of mother’s colostrum lays the foundation for life-long immunity. In some cases, the neonate’s immunity is compromised due to the lack of mother’s colostrum or breast-feeding difficulty (Le Doare et al., 2018). Consequently, GID problems arise during adolescence or adulthood due to a deficient immune system. It is therefore imperative for neonates to consume colostrum for physical growth and proper development of the immune system, and to curb GID disorders later in life.

It has been suggested that bovine colostrum (BC) contains almost ninety bioactive components. These bioactive substances consist of immunoglobulins and growth factors, antibodies, higher levels of amino acids, oligosaccharides, antimicrobial compounds, and immune regulators like lactoferrin (Jacqmin, 2000). BC is also rich in vitamins and minerals. Colostrum provides nutrients in a highly concentrated low-volume form to the newborn. Due to its laxative properties, colostrum assists in the passage of baby’s initial stools or meconium and helps to remove excess bilirubin from the infant’s body to prevent jaundice (Buttar et al., 2017). Excess accumulation of bilirubin in the neonate can cause jaundice, anemia, liver cirrhosis, and Gilbert’s syndrome (Maqbool, 1992; Crittenden et al., 2007; de Almeida and Draque, 2007). Research has shown that BC is 100-fold to 1,000-fold more potent than human colostrum. Thus, human infants can thrive very well by consuming infant- formula containing BC supplements that can provide passive immunity and growth factors needed for physical and gastrointestinal development. BC is an emerging nutraceutical and innovative therapeutic products being developed for children and adults.

### Cancer

Cancer is the second leading cause of mortality and morbidity worldwide, behind only cardiovascular diseases (Siegel et al., 2016; Miller et al., 2019). Cancer is a collective name for a disease where the abnormal body cells divide in an uncontrollable fashion in a body part or organ, resulting in a tumor or carcinoma. Genetic, epigenetic, and environmental factors play an important role in the occurrence and progression of cancer. There are two types of cancer: benign or noninvasive and malignant or invasive. Malignant cancer cells can invade nearby tissues or organs and can also travel to distant places in the body through blood or the lymphatic system, and consequently form a new tumor far away from the original one. According to Seebacher et al. (2019), nearly 14.1 million people suffer from cancer, and about 9.6 million deaths were reported worldwide in 2018. From a mortality point of view, 1 out of 6 deaths are caused by cancer (Bray et al., 2018). The common types of cancers include: carcinoma, sarcoma, leukemia, lymphoma, melanoma, lung, colorectal, prostate, and breast cancer (Brandal and Heim, 2015; Linehan et al., 2016; Rosenberg et al., 2016; Tricoli et al., 2016; Bullinger et al., 2017; Etienne et al., 2017; Meurer et al., 2017; Rajyaguru et al., 2018; Miolo et al., 2019). The current strategies for cancer treatment consist of chemotherapy, radiotherapy, bone marrow transplants, and surgery. However, these therapies have drawbacks and limitations; for instance, radiation therapy causes indirect damage to surrounding tissues, and chemotherapy results in vital organ toxicity and also causes drug resistance, whereas surgical interventions may sometimes precipitate tumor recurrence (Formenti and Demaria, 2009; Taylor and Kirby, 2015; Vitetta et al., 2019). Recent trends in cancer treatment also include targeted drug delivery and immunotoxin therapy (Vitetta et al., 2019). Immunotoxin is a conjugated protein which blends a targeted conjugate with a toxin. These immunotoxins enter into the cancer cell through endocytosis and lead to cell death.

Stomach, colorectal, and lung cancer are common in both sexes, whereas liver and prostate cancer is common in men, and breast and cervical cancer occur in women. Currently, gastric cancer is one of the serious diseases worldwide. According to global cancer statistics data, gastric cancer is the fourth most common cancer worldwide. Serious vital organ deleterious effects happen when gastric cancer is treated by chemotherapy (Rugge et al., 2015; Rathe et al., 2019). Therefore, there is an urgent need for the development of less toxic therapeutic agents for the prevention and cure of stomach cancer (Farziyan et al., 2016).

### Role of BC for the Treatment of Cancer and Gastrointestinal Diseases (GIDs)

Colostrum or first milk is secreted by all female mammals, including women, during the first four days after parturition and is provided to their neonates during the initial 24-48 hours after birth (Bagwe et al., 2015; Agarwal and Gupta, 2016; Hyrslova et al., 2016; Jolly and Mascaro, 2016; Buttar et al., 2017). Colostrum is thick, sticky, yellowish liquid which not only provides nutrition and immunity but also gives protection against microbial infections. Almost all essential nutrients such as protein, fat, lactose, lactoferrin, immunoglobulins, vitamins and minerals, and growth factors are present in the colostrum in significantly higher concentrations than the regular mature milk (Shah, 2000). Colostrum creates life-long immunity in the newborn and helps in maturing the GI tract of babies. Whereas in adults, colostrum promotes healing of the GI tract and protects against gut pathogens (bacteria, viruses, fungi, yeast, mold, etc.), and leaky gut syndrome (Shah, 2000; Hurley and Theil, 2011; Osada et al., 2014; Wu Xiaoyun and Xiong Lin, 2015). The major bioactive components of bovine colostrum and their functions in children and adults are summarized in **Table 1**. BC acquired from cows and buffalo possess more immunoglobulins than human colostrum, and human infants could benefit by consuming BC (Ulfman et al., 2018). BC is usually regarded as safe in humans, whereas some people may experience nausea and flatulence initially, which declines over time. BC should not be given to individuals allergic to milk and milk products.

**Table 1:** Composition of bovine colostrum and functions in children and adults.

Components	Function	Reference
Vitamins (A, B1, B2, B6, B12, D, E) Minerals (Na, K, Ca, P, S, Mg, Mn, Zn, Cu, Fe) Amino acids and essential fatty acids	Promote health and growth of the infant.	(McGrath et al., 2016)
Immune factors Proline-rich polypeptide (PRP) Immunoglobulins Lactoferrin	Regulate function of the thymus gland, and reduce oxidative stress. IgG neutralizes toxins and microbes. IgA, IgD, IgE, and IgM destroy bacteria and are highly antiviral. Lactoferrin is an anti-viral, anti-inflammatory, and anti-bacterial iron-binding glycoprotein with potential therapeutic applications in cancer and HIV.	(Stelwagen et al., 2009)
Growth factors Growth hormone (GH) Platelet-derived growth factor (PDGF) Fat (6.7%) Protein (14.9%) Lactose (2.5%)	Stimulate DNA synthesis, enhance cell growth and tissue growth.	(Stelwagen et al., 2009)

N. B. Fat content in cow colostrum (6.7%) is higher than human colostrum (3**–**5%).

Protein content in cow colostrum (14.9%) is significantly greater than human colostrum (0.8–0.9%).

Lactose content in cow colostrum (2.5%) is significantly lower than human colostrum (6.9–7.2%).

[Source: (Bagwe et al., 2015; Buttar et al., 2017)]

The central theme of this review is to address the potential benefits of colostrum nutrients in children and adults as well as the usefulness of BC components for the treatment of cancer and gastrointestinal disorders. As discussed earlier, the conventional cancer therapies include chemotherapy, radiotherapy, bone marrow transplant, and surgical interventions, but these therapies have drawbacks and limitations. Hence there is a need for cost-effective and safe novel therapies for the treatment of cancer. Limited numbers of clinical trials with BC have shown anticancer effects in different cancer types (Layman et al., 2018; França-Botelho, 2019). The anticancer effects of lactoferrin, proline rich polypeptides, conjugated linolenic acid (CLA), and alpha-lactalbumin are presented in **Table 2**. The nutrient profile of BC is markedly different from mature milk. The quantitative concentrations of the main constituents of cow colostrum and cow milk are depicted in **Table 3**. It can be seen from **Table 4** that the concentrations of lactoferrin, IgA, insulin like growth factor, growth hormone, and epidermal growth factor are markedly higher in human colostrum as opposed to bovine colostrum (Godhia and Patel, 2013, Bagwe et al., 2015).

**Table 2:** Important components of bovine colostrum: Functions and anticancer effects.

Component	Function	Anticancer Effect	Reference
Lactoferrin	Antibacterial, antiviral, antitumor	Gastric, lung, colorectal, breast	(Layman et al., 2018; França-Botelho, 2019)
Proline-rich polypeptides, Conjugated linolenic acid (CLA)	Promotion of T and NK cell activation	Ovaria, breast, rectal	
Alpha-lactalbumin	Antiviral, antitumor	Breast	

**Table 3:** Main components of bovine colostrum and bovine milk: Amounts represented as per liter.

Component	Bovine colostrum(per liter)	Bovine milk(per liter)	Reference
IgG1	35-90 gram	0.30-0.40 gram	(Elfstrand et al., 2002) (Bagwe et al., 2015) (Buttar et al., 2017)
IgG2	1.5-2 gram	0.03-0.08 gram	
IgA	3-6.5 gram	0.04-0.06 gram	
IgM	3.8-6 gram	0.03-0.06 gram	
Lactoferrin	1.5-5 gram	0.1-0.3 gram	
Crude protein	41-140 gram	34 gram	
Growth hormone (GH)	<1μg	<0.03μg	
TNF-α	926 μg	3.3μg	

**Table 4:** Comparison of human colostrum and bovine colostrum.

Component	Human colostrum (mg/ml)	Bovine colostrum (mg/ml)	Reference
Lactoferrin	700	100	(Godhia and Patel, 2013) (Bagwe et al., 2015)
IgA	17.35	3.9	
IgG	0.43	47.6	
IgM	1.59	4.2	
Insulin-like growth factor	18	10	
Growth hormone	41 ng/L	<0.03ng/L	
Epidermal growth factor	200 μg/L	30-50 μg/L	

## Role of Human and Bovine Colostrum in the Maturation of GI Tract in Babies

Many researchers have shown that colostrum plays a critically significant role in the growth and maturity of the GI tract in infants. The nutrients in colostrum create a suitable environment - namely biochemical, physiological, morphological, functional, immunological, and antimicrobial - for the maturity of the gastrointestinal tract in new-born babies (Pluske, 2016). Recent studies performed on piglets, serving as a model for human infants, have suggested that the epidermal growth factor of BC is responsible for the growth and maturity of the GI tract in infants (Bedford et al., 2015). Another study in piglets also demonstrated the growth promoting effects of bovine lactoferrin in the stimulation of intestinal cell proliferation, increased crypt depth, and villus length. Lactoferrin is a glycoprotein with strong antioxidant, anti-inflammation, anti-cancer, and anti-microbial properties. Lactoferrin induces the stimulation of T-helper-1/T-helper-2 cytokine immune response and secretion of anti-inflammatory cytokines. It has been observed that lactoferrin can prevent gastric infections, necrotizing enterocolitis and late onset sepsis in children (Pammi and Abrams, 2015; Donovan, 2016; Pieper et al., 2016).

## Application of BC in Treating Inflammatory Bowel Disease (IBD) and Nonalcoholic Steatohepatitis (NASH)

Inflammatory bowel diseases (IBDs) result from alterations in the systemic immune response and modulation of the gut immune system, which induce inflammation-mediated damage to the gastro-intestinal tract and injury to related organs. BC supplements have been used as an alternative therapy for the treatment of nonalcoholic steatohepatitis (NASH) and insulin resistance type 2 diabetes and colitis. Hyperimmune bovine colostrum is enriched with IgG and enhanced with glycosphingolipid immune adjuvants and anti-lipopolysaccharides. To determine the safety and efficacy of hyperimmune bovine colostrum (Imm124-E), Mizrahi et al. (2012) performed an open-label trial in ten patients diagnosed with insulin resistant type 2 diabetes and nonalcoholic steatohepatitis (NASH). Oral administration of Imm124-E at doses of 600 mg thrice daily (1800 mg/day) for 30 days improved type 2 diabetes and hyperlipidemia, and alleviated NASH through immunomodulatory action without any adverse effects. Oral administration of Imm124-E to mice ameliorated immune-mediated colitis induced by intra-colonic instillation of trinitrobenzene sulfonate. Imm124-E improved bowel histology and regeneration score, and decreased the extent of colitis damage in mice. This pathophysiological improvement was associated with the elevation of serum IL10, anti-inflammatory cytokine levels, CD4+, CD25+, and CD4+ Foxp3+ Tregs (Ya’acov et al., 2015). According to Ilan (2016), oral immune modulation therapies (e.g. nutraceuticals, functional foods, probiotics, prebiotics, polyunsaturated fatty acids, polyphenols, non-absorbable gut-associated adjuvuants, etc.) may be helpful to re-establish gut tolerance and to alter the gut immune system *via* the modulation of intestinal microbiota to treat autoimmune and inﬂammatory disorders like IBD.

## Application of BC Supplements in Treating Crohn’s Disease and Gut Infections

Colostrum may be beneficial in chronic inflammatory diseases, such as various forms of arthritis, Crohn’s disease, or inflammatory bowel disease (IBD). Crohn’s or Celiac disease is an inflammatory bowel disease that causes abdominal pain and diarrhea (Sequeira et al., 2014). Non-steroidal anti-inflammatory drugs (NSAIDS) are often prescribed to reduce pain and abdominal cramps. However, chronic use of NSAIDS can cause peptic ulcers and alterations of gut microbiota, and the latter may induce leaky gut syndrome. BC possesses strong anti-inflammatory and anti-bacterial effects and can neutralize the lipopolysaccharides produced by gram negative bacteria (Rawal et al., 2008). BC reduces the expression of TNF-α in Caco-2 and HT29 cell lines as well as inhibits IL-8 expression and production of inflammatory cytokines, and consequently reduces gut inflammation. BC also decreases the adherence of invasive E. coli bacteria in human cell lines (Chae et al., 2017). Collectively, these findings suggest the promising therapeutic potential of BC in treating GI tract infections and inflammation-related IBD. Results of clinical and preclinical studies done with BC and dosage forms used for curing internal pathologies and external wounds are shown in **Table 5**.

**Table 5:** Colostrum dosage forms used for treating internal pathologies and external wounds.

Types of Injuries	Colostrum dosage form	Number of patients enrolled in clinical trials (Male/Female)	Endpoints monitored	Effect observed	Dose and study duration	Reference
Gastrointestinal injury due to non-steroidal anti-inflammatory drugs	Colostrum powder in the form of tablets/capsules.	7 (7/0)	Intestinal permeability	IGF and TGF-β responsible for analgesic activity	125 ml t.i.d. for 7 days	(Playford et al., 2001)
Inflammation induced for HIV patients for infection in gastroesophageal tract.	Colostrum powder in the form of tablets/capsules or liquid colostrum.	87 (27/60)	Stool frequency; self-reported fatigue; CD4^+^ count; body weight	Mucosal integrity, tissue repair, and antimicrobial actions	16 G b.i.d. for 4 weeks	(Kaducu et al., 2011)
Diabetes Delayed injury healing due to increase in blood glucose levels	Colostrum topical cream or colostrum powder in the form of tablets/capsules.	18 (9/9)	Postprandial blood glucose; triglycerides; cholesterol; ketone bodies	Reduction of blood glucose, which starts wound healing problem in diabetic patients.	10 G daily	(Kim et al., 2009)
Repair of muscle, bone tissue, skin cartilage, and nerve cells.	Colostrum topical cream or colostrum powder in the form of tablets/capsules	No clinical studies were done in humans; only *in vitro* cell line studies were done.	Increases the migration of WI38 fibroblasts	Nucleotides, epidermal growth factor, transforming growth factor, and IGF-1 promote wound healing and DNA, RNA damage repair.	10 G per 100 G of cream composition	(Takayama et al., 2001)
Ultraviolet B (UVB)-induced photo damage	Colostrum topical cream	Pre-clinical studies done on seven-week-old male Hos : HR-1 hairless mice.	Trans-epidermal water loss starts to increase.	Lactoferrin capable of preventing damage to the skin	10 G per 100 G of the cream composition	(Murata et al., 2014)
Infection with diarrhoeic *E. coli* in children	Colostrum powder in the form of tablets/capsules	27 (13/14)	Stool frequency; elimination of strainsexpressing virulence factors	Decrease stool frequency	21 G once daily for 14 days	(Huppertz et al., 1999)
Chronic pain syndrome	Colostrum powder in the form of tablets/capsules	4(2/2)	Flow cytometry; cytokine analysis;IGF-1; apoptosis	Apoptotic effect on monocytes	20 G once daily for 14 days	(Waaga-Gasser et al., 2009)
Inflammatory bowel disease (IBD)	Colostrum enemas	14(6/8)	Mild-to-moderate severe distal colitis (IBD), histological score was used for clinical assessment	Improvement in histological scores showed reduction of IBD symptoms.	100 ml of 10% BC solution enemas b.i.d	(Khan et al., 2002)

## Role of BC and Components in Treating Gut Diseases Caused by Microbial Infections

Generally, acute infectious diarrhea, immunodeficiency diarrhea, short bowel syndrome, IBD, etc. are treated with synthetic pharmaceuticals (Holtmann et al., 2017). The secondary infections in the GI tract and diarrhea in AIDS patients are also treated with drugs (Playford et al., 1999). A limited number of randomized, double-blind, and controlled studied have been done in children and adults to evaluate the efficacy of BC supplements for treating gut diseases caused by microbial infections. BC and its components were found to be effective against Gram negative and Gram positive bacteria and helped in treating gut infections and diarrhea. The dosages of BC supplements and bioactive ingredients used in preclinical and clinical studies for treating gastrointestinal diseases are summarized in **Table 6**.

**Table 6:** Pre-clinical and clinical applications of bovine colostrum in gastrointestinal diseases.

GI tract disease	Preclinical and clinical manifestations	Preclinical/Clinical studies					Reference
		Study Design	No. of patients enrolled in clinical trials (BC/placebo)	Endpoints observed	Effect	Dose	
Acute infectious diarrhea	Eliminate pathogen, improve the intestinal barrier function, inhibit bacterial translocation, and reduce disease severity	Double-blind randomized-controlled trial	160 children (80/80)	Investigated for bacterial/viral causes of diarrhea (*Salmonella, Shigella, E. coli, Campylobacter and Vibrio choler*a; Rotavirus antigen in stool)	Lower frequency of vomiting and diarrhea	3G/sachet in 50 ml ordinary water	(Menchetti et al., 2016) (Barakat et al., 2020)
*Helicobacter pylori* infections	Inhibit the invasive capacity of pathogen bacteria, modulation of immune response, and favor mucosal repair	Randomized- controlled trial	C57BL/6 female mice subjected to 0.1 ml of 1×10(9) *H. pylori*	Bacterial load, gastric emptying time	Increased gastric emptying time (7.9 min)	0.1ml HNZ (hyperimmune bovine colostrum plus N-acetyl cysteine plus zinc)	(Rokka et al., 2008; Tran et al., 2010; Xu et al., 2015)
Immunodeficiency diarrhea	Reduced abdominal pain, diarrhea score, and fatigue, reduced daily stool frequency, and increased the body weight and body mass index	Randomized, single-blind trial	84 Adults (ColoPlus^®^/placebo) (43/41)	Daily stool frequency, body weight, body mass index, and baseline CD4^+^ count	Mean daily stool frequency (↓79%), self-reported fatigue (↓85%), mean CD4^+^ count (↑14%)	50 G, twice daily	(Florén et al., 2006; Kaducu et al., 2011)
Short bowel syndrome (SBS)	Support intestinal development and function in newborn, and also enhance intestinal adaptation and functions	Randomized, double-blind, crossover, pilot study	9 children	Intestinal absorption of energy and wet weight	No improvement in the wet weight or intestinal absorption	20% of the children’s basal fluid requirement (BFR)	(Ausholt et al., 2014; Støy et al., 2014; Shen et al., 2015)
Inflammatory bowel disease (IBD)	Reduced weight loss, decreased colon shortening, and improved the histologic severity of colon inflammation	Randomized- controlled trial	Six-week-old CD-1 mice	Clinical signs, histopathological characteristics, expression levels of toll-like receptor 4 (TLR4), pro- and anti-inflammatory cytokines, and microbial composition	↓TLR4 (p < 0.01), ↓Interleukin-1β (IL-1β; p < 0.001), ↓Interleukin-8 (IL-8; p < 0.001), and ↓Interleukin-10 (IL-10; p < 0.001)	100 mg of colostrum powder dissolved in 0.6 ml of physiological saline solution was given to each mouse	(Bellavia et al., 2014) (Menchetti et al., 2020)
Necrotizing enterocolitis (NEC)	Maturation of the digestive tract, balance gut microbiota, modulation of the intestinal immune system, and mucosal repair	Randomized-controlled trials	Four trials, 678 participants	Neonatal sepsis and necrotizing enterocolitis (NEC)	Late-onset sepsis (risk ratio (RR) 0.49, 95% confidence interval (CI) 0.32 to 0.73) NEC ≥ stage II RR 0.3 95% CI (0.12 to 0.76)	Oral lactoferrin 200mg/day	(Cairangzhuoma et al., 2013; Good et al., 2015);

## Role of Lactoferrin and Lactalbumin in Cancer Therapy

Lactoferrin (LF) is an excellent immune modulator and anticancer agent and has a tissue regenerative capacity. It can also inhibit the production of inflammatory cytokines. Lactalbumin is present in whey and can markedly improve the immune response and enhance the synthesis of glutathione. It has been observed that lactoferrin and lactalbumin can induce apoptosis in cancerous cells (Teixeira et al., 2019). LF has been reported to elevate the level of caspase-1 and IL-18, and in turn reduce the metastatic foci in the intestine. LF-induced apoptotic activity of cytotoxic T and natural killer (NK) cells has also been observed. In addition, LF inhibits hepatic CYP1A2 enzyme, which is responsible for the activation of carcinogens (Tsuda et al., 2006). LF may be employed as a carrier for chemotherapeutic agents, especially for the treatment of brain tumors, due to its ability to cross the blood-brain barrier (Cutone et al. (2020). It therefore appears that LF and whey lactalbumin can be used as combination adjunct therapies with chemo- and radiotherapy for treating cancer. This approach would not only enhance the chemotherapeutic effectiveness of drugs, but also limit the use of chemo- and radiotherapy, resulting in reduced incidences of undesirable side effects observed in cancer patients.

## *In Vitro* Evaluation of the Anticancer Effects of BC Components Using Different Human Cancer Cell Lines

*In vitro* cell culture studies are used in selected cancer cell lines as a promising tool to determine the antiproliferative and cytotoxic effects of potential anticancer agents isolated from natural sources or synthesized in the laboratory. *In vitro* cell culture studies provide clues about the mechanism of action of anticancer agents toward cancer cells. Anti-cancer effects of lactoferrin were evaluated using MTT assay. The addition of lactoferrin in the culture medium inhibited the growth of cancer cell lines (MDA-MB-231 and MCF-7) (Sharma et al., 2019). Purified lactoferrin (2 mg/ml) retarded the growth of esophageal cancer cell lines (KYSE-30) and HEK cancer cell lines. The addition of 500 µg/ml of lactoferrin in the culture medium decreased the cell viability of KYSE-30 cancer cells by 80% after 62 hours’ exposure. No effect was noted in the normal HEK cell line. Flow cytometry analysis suggested that lactoferrin induced apoptosis in KYSE-30 human esophagus cancer cell lines (Farziyan et al., 2016). The results of *in vitro* studies done to assess the anticancer properties of BC components (lactoferrin, liposomal bovine lactoferrin, bovine lactoperoxidase, lactoferrin nanoparticles, and conjugated linolenic acid) on different cancer cell lines (e.g., gastric, esophagus, colorectal, liver, lung, prostate, breast, ovarian) are summarized in **Table 7**.

**Table 7:** Anticancer effects of bovine colostrum components on different cancer cell lines.

Component of bovine colostrum	Cancer type	Dose	Result	Reference
Lactoferrin	Gastric cancer (AGS human stomach carcinoma cell)	500 μg/ml	Caused 80% cytotoxicity in AGS cell line	(Amiri et al., 2015)
Lactoferrin	Human esophagus cancer cell (KYSE-30 esophageal squamous cell carcinoma)	500 μg/ml	Inhibited the development of azoxymethane (AOM)-induced aberrant crypt foci (ACF) by 53% and 80% after 20 and 62 h, respectively.	(Farziyan et al., 2016)
Liposomal bovine lactoferrin	Colorectal cancer (RKO and RCN-9 human CRC cells)	≥10 μg/ml	Significant inhibition of colon aberrant crypt foci growth occurred in the RKO and RCN-9 cells (P<0.01).	(Sugihara et al., 2017)
Bovine lactoperoxidase (LPO) and lactoferrin (LF) nanoparticles	Colorectal cancer (Caco-2), liver cancer (HepG-2), breast cancer (MCF-7), prostate cancer (PC-3).	315-1388 μg/ml	Ten-fold suppression in NF-kB expression in Caco-2, HepG-2, and MCF-7; four-fold downregulation of NF-κB mRNA level in PC-3 cell lines; 15-fold decrease in Bcl-2 levels, as compared to treatment with 5-flurouracil.	(Abu-Serie and El-Fakharany, 2017)
Lactoferrin	Lung cancer (human lung cancer cell line, A549)	0.9375-15 mg/ml	Decreased expression of *VEGF* mRNA and VEGF protein in a concentration-dependent manner.	(Tung et al., 2013)
Conjugated linolenic acid (CLA)	Ovarian cancer cells (SKOV-3 and A2780 cells)	7 μM CLA for 48 to 72 h	Nine-fold increase in autophagolysosomes, G1 cell cycle arrest in SKOV-3 and A2780 cell lines by downregulation of E2F1.	(Shahzad et al., 2018)
	Breast cancer cell line (MCF-7), colon cancer cell line (HT-29), (mouse fibroblast cell line Balb/3T3)	0.1-100 µg/ml	Reduced anti-apoptotic Bcl-2 expression	(Niezgoda et al., 2017)

## *In Vivo* Anticancer Effects of BC Components in Animal Models and Humans

**Figure 1:**
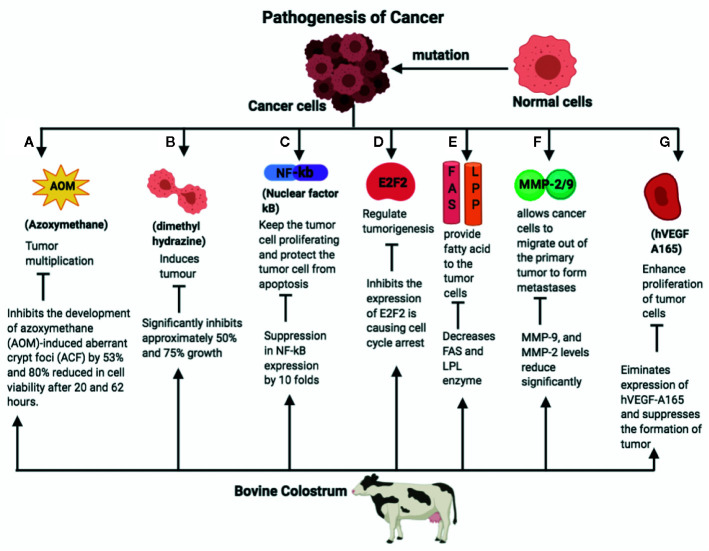
A normal cell mutates into the cancer cell, which divides enormously and spreads across the surrounding tissues. The pathways responsible for initiation and metastasis of cancer and the role of bovine colostrum (BC) on the amelioration of the same is given as follows. **(A)** Azoxymethane is responsible for tumor multiplication and cancer cell development. BC inhibits the development if azoxymethane (AOM)-induced aberrant crypt foci (ACF), therefore reducing the cell viability. **(B)** BC inhibits the proliferation of tumor angiogenesis by dimethylhydrazine. **(C)** Nuclear factor-κB (NF-κB) is the transcription factor that is responsible for cancer cell growth, tumor formation, and tumor cell proliferation, and prevents tumor cells from apoptosis. BC suppresses NF-κB expression approximately by 10-fold. **(D)** E2F2 factor plays an important role in the tumorigenesis of the cancerous cells. BC inhibits expression of E2F2 factor and initiates cell cycle arrest. **(E)** BC inhibits the enzymes fatty acid synthetase (FAS) and lipoprotein lipase (LPL), which in turn inhibit neoplastic lipogenesis. **(F)** Matrix metalloproteinaise 2/9 (MMP-2/9) allows cancer cells to migrate out of the primary tumor to form metastases. BC supresses the levels of the MMP- 2/9. **(G)** BC inhibits the expression of Human Vascular Endothelial Growth Factor (hVEGF), which is responsible for proliferation of the cancer cells. AOM, Azoxymethane; ACF, Azoxymethane induced aberrant crypt foci; NF-κB, Nuclear factor Kappa B; E2Fi, E2F Transcription Factor 1; FAS, Fatty acid synthetase; LPL, Lipoprotein lipase; MMP, Matrix metalloproteinase; hVEGF, Human Vascular Endothelial Growth Factor.

Following the information obtained from *in vitro* studies, the next step involves preclinical investigations in appropriate animal models to assess the safety, efficacy, and toxicity of anticancer agents. Numerous anticancer studies with BC supplements and major components have been done on rodents. For instance, lactoferrin and conjugated linolenic acid (CLA) have been tested for treating colorectal, lung, and esophageal cancers in rats and mice. Reduction in colon tumor load and downregulation (**Figure 1**) in the expression of VEGF were observed in the preclinical studies (Tung et al., 2013; Sugihara et al., 2017). A limited number of clinical trials in a small number of patients have been performed in humans to understand the anticancer potential of BC components. Based on the promising anticancer effects of CLA in preclinical models, an open-label clinical study was done on 24 women diagnosed with breast cancer. CLA was given orally at doses of 7.5 G/day for 20 days. CLA was found to suppress the expression of fatty acid synthase (FASN) and lipoprotein lipase (LPL). The depressed activity of these biomarker enzymes indicates the suppression of breast tumor growth (McGowan et al., 2013). The results of another clinical trial suggested that CLA (3G/day) may be useful in rectal cancer patients undergoing chemoradiotherapy (Mohammadzadeh et al., 2013). The information obtained from preclinical and clinical effects of lactoferrin, liposomal lactoferrin, and CLA in different types of cancers is shown in **Table 8**.

**Table 8:** Preclinical and clinical effects of bovine colostrum components on different cancer types in animals and humans.

Components of bovine colostrum	Cancer type	Dose	Preclinical/clinical study results	Reference
Liposomal Lactoferrin	Colorectal cancer (Thirty-six F344 male rats, 5-weeks-old, were used in the experiment).	1,000 mg/kg/day in drinking water	Approx. 43% reduction was observed in the colon tumor.	(Sugihara et al., 2017)
Lactoferrin	Lung cancer (12 transgenic mice)	300 mg/kg, 3 times a week.	Significantly decreased expression of hVEGF-A_165_ and suppressed the formation of tumor.	(Tung et al., 2013)
Conjugated linolenic acid (CLA)	Breast cancer (24 women)	7.5 G/day in the form of capsules for 20 days	Decrease in FAS and LPL enzymes which provide fatty acids for breast tumor growth.	(Kuemmerle et al., 2011; McGowan et al., 2013; Arab et al., 2016)
	Rectal cancer (33 human volunteers)	3G/day in the form of capsules for six weeks.	Significant changes occurred in TNF-α (P = 0.04), hsCRP (P = 0.03), and MMP-9 (P = 0.04)	(Mohammadzadeh et al., 2013)

## Intravaginal Application of BC Tablets Cause Spontaneous Regression Of HPV-Associated Low-Grade Cervical Intraepithelial Lesions in Women

Human papillomavirus (HPV) infection is the most common sexually transmitted disease in young people worldwide. Most infections are cleared by the immune system, but persistent infections may cause intraepithelial abnormalities in the infected cells that can develop into cancers of the cervix, vagina, vulva, anal canal, and penis. Immunotherapy is considered the most promising treatment for HPV-related pathologies. HPV vaccines have been developed to prevent HPV-associated cancer, external genital lesions, and genital warts (Bergman et al., 2019). In Italy, the immunomodulating action of BC was evaluated in an observational, multi-centre, pilot study, where 256 patients were enrolled with a history of low-grade cervical squamous intraepithelial lesions. At baseline, all patients were tested for cervical cytology (Pap smear), colposcopy, and targeted biopsy. BC-containing vaginal tablets (GINEDIE^R^) were administered twice a week at bedtime for 24 weeks, without any other medication for the whole study period. The rates of regression were recorded histologically at the end of the study period. Overall regression rate with negative histology was 75.5% at the end of the 6 month follow-up period. The patients did not experience any adverse effects during the treatment. The authors concluded that, as opposed to a spontaneous regression period of 1-5 years, intravaginal topical application of BC significantly shortens the regression time of low-grade cervical intraepithelial lesions to half a year (Stefani et al., 2014).

## Current and Future Developments of BC Nutraceuticals

In this review, we have attempted to summarize the nutraceutical health benefits of BC, and the therapeutic potential and effectiveness of marketed colostrum powder, capsules, and tablets for the treatment of various types of cancers and GI tract pathologies. BC is an emerging nutraceutical and innovative therapeutic products are being developed for children and adults. In future, BC products could be a boon in providing non-hazardous, cost-effective, and affordable alternative sources of natural remedies for treating different types of cancers, GIDs, and autoimmune disorders. However, there are several challenges and opportunities that need to be addressed. For instance, well-designed, placebo-controlled, and randomized clinical trials are needed to determine the long-term safety, effectiveness, and optimal doses of BC supplements. Some other aspects of BC nutraceuticals include the standardization of products originating from different breeds of cows and buffalo. In addition, good manufacturing practices and standardized techniques are required for making BC formulations, and possible adulteration of BC supplements with synthetic drugs and microbial contaminants, just to name a few. More basic research is needed to understand the mechanism of action of different components of BC for their anticancer and antidiabetic properties, and for curing wounds, gastrointestinal disorders, and inflammatory bowel diseases.

Nutraceuticals are defined as substances that provide physiological benefits and assist in improving overall health beyond basic nutritional functions and protect against non-communicable diseases. Generally, nutraceuticals consist of products isolated or purified from vegetables and fruits, colostrum supplements, and dairy products, and are sold as non-pharmacological, cost-effective, and affordable alternative therapies for the prevention and treatment of neurodegenerative and cardiovascular diseases, musculoskeletal abnormalities, diabetes, obesity, and some cancers. The influence of nutraceuticals, functional foods, natural health products, dietary supplements, and probiotics is often neglected by healthcare professionals and leading experts in the field of medicine. Nutraceuticals could be one of the biggest drivers for curing the global epidemic of chronic non-communicable diseases, including obesity, diabetes, cardiovascular diseases, and certain cancers. However, the evidence-based dietary advice is beset by poor quality science, a limited number of randomized, placebo-controlled studies, and unresolved controversy about the role of nutraceuticals in curing non-communicable diseases. Good manufacturing practices (GMPs) and high-quality control standards should be used for the manufacturing of BC nutraceuticals. Post-marketing surveillance should be conducted diligently for the tolerability of BC supplements and bioactive components. Dairy farmers should be encouraged to collect BC using sterile and hygienic conditions as much as possible.

Bovine colostrum is significantly rich in biologically active peptides, antioxidants, anti-inflammation agents, and growth promoting factors that differ substantially from later milk. The benefits of BC are well known in the health and disease of children and adults. As discussed earlier, BC is an emerging nutraceutical and innovative therapeutic products that is being developed for children’s formulas and for the treatment of non-communicable diseases. Hopefully, BC supplements will greatly contribute to curing different cancer types, diabetes, cardiovascular diseases, necrotizing enterocolitis, and inflammatory bowel disease or Crohn’s disease, and autoimmune disorders. Understanding the biological roles of different BC ingredients is a major challenge for nutritionists and dieticians, basic researchers, and physicians.

BC can also mitigate a wide variety of bacterial, viral, fungal, and parasitic infections. BC impregnated dressings are non-allergic, safe, and promote wound-healing. Such dressings may be used as an adjunct for the management of deep wounds and burns. BC is richer in immunoglobulins than human colostrum and can be used in the treatment of immunodeficiency diseases and infections along with conventional medicines (Bagwe et al., 2015; Buttar et al., 2017). Our studies indicated that BC possesses strong antimicrobial activity against both Gram^-tive^ and Gram^+tive^ strains. The minimal inhibitory concentration (MIC) of colostrum was found to be 100 µg/ml against *E. coli, S. aureus, P. vulgaris, E. aerogenes, and S. typhi* (Yadav et al., 2016). It is possible that BC might have viricidal effects against the COVID-19 virus. Lactoferrin especially is well known for its anti-inflammation and anti-microbial properties. This hypothetical idea may be worth pursuing!

## Conclusions

BC supplements have proven useful in the management of GIDs, such as acute infectious diarrhea, Helicobacter pylori infections, irritable bowel syndrome, inflammatory bowel disease (IBD), and different types of human cancer cell lines (e.g. esophagus, colorectal, lung, breast and ovarian cancer). The components of BC, such as lactoferrin, CLA, and alpha-lactalbumin, are useful in treating GI-related disorders and some cancer types. The oral consumption of BC can boost the immune system and improve the inflammatory condition of patients suffering from gastrointestinal disorders.

BC possesses strong antibacterial, antiviral, and antifungal properties, and has also exhibited antitumor actions in a limited number of *in vitro* and *in vivo* studies. Several components of BC have shown apoptosis in cancer cells and suppression in the growth of tumors. Also, NK cells are inhibited after BC exposure. While BC products are well tolerated, some patients allergic to dairy products may experience undesirable side effects. Overall, BC supplements can be safely used for the treatment of GIDs, autoimmune disorders, and different cancers. The clinical interactions of BC, if any, with orally administered prescription or over-the-counter drugs should be explored regarding the bioavailability and pharmacokinetics, and the possibility of such an interaction should be monitored in patients using synthetic drugs for co-morbid conditions.

## Author Contributions

GK, HS, and HT visualized the presented idea, contributed to manuscript writing, and supervised the project. SB-P and PY contributed to literature searches and to preparing the manuscript draft. GK and HS revised and approved the manuscript.

## Conflict of Interest

The authors declare that the research was conducted in the absence of any commercial or financial relationships that could be construed as a potential conflict of interest.

## Abbreviations

BC, Bovine colostrum; CLA, Conjugated linolenic acid; GID, Gastrointestinal disorders; GIT, Gastrointestinal tract; IBD, Inflammatory bowel disease; NASH, Non-Alcoholic Steato Hepatitis; NK, Natural killer; NSAIDS = Non-steroidal anti-inflammatory drugs.
